# Special Issue “Dietary Bioactive Components in Inflammatory Bowel Disease”

**DOI:** 10.3390/ijms25073569

**Published:** 2024-03-22

**Authors:** Massimiliano Gasparrini, Luca Mazzoni

**Affiliations:** Department of Agricultural, Food and Environmental Sciences, Università Politecnica delle Marche, Via Brecce Bianche 10, 60131 Ancona, Italy; m.gasparrini@staff.univpm.it

Inflammatory bowel diseases (IBD) comprise chronic debilitating inflammatory disorders that can affect different parts of the gastrointestinal tract and are commonly correlated to two main diseases: Crohn’s disease (CD) and ulcerative colitis (UC). Anemia, malnutrition, infection, and an increased risk of the development of colon cancer represent the severe complications of these inflammatory conditions. The etiology of IBD is not well established; the interaction of multiple factors, including environmental and genetic factors, intestinal microbiota, and immune, response seems to be involved in IBD promotion. The prevalence and incidence of IBD increased remarkably during the second half of the 20th century and since the beginning of the 21st century, in newly industrialized countries (Europe and North America). In addition, considering that there is no cure for IBD, it is becoming very important to find new tools that could efficiently counteract the onset and development of this disease. In this sense, phytochemicals and other dietary bioactive compounds mainly present in fruits and vegetables represent nutritional supplements for the prevention and treatment of IBD, considering their consolidated and widely investigated anti-inflammatory and anti-oxidative effects. The goal of our Special Issue was thus to investigate the recent advances in the study of the role exerted by dietary bioactive compounds against IBD, focusing on the cellular processes, molecular mechanisms, and pathways involved to elucidate their possible beneficial health effects and to promote their efficacy in disease management.

Seven papers have been accepted and published from January 2022 to February 2023: five research articles and two reviews. Among these, both in vitro and in vivo studies (mice and human) have been conducted, mainly on three topics related to IBD: the effect of natural bioactive compounds against UC, CD, and colorectal cancer (CRC). Three out of the five research articles in this collection deal with the in vivo effect of dietary components on CD, but with different approaches: in vivo studies on animal models (in two of the papers) and cohort study on the human population (in one). Regarding the first approach, Kopiasz et al. [1] investigated the molecular mechanisms underlying the anti-inflammatory properties of oat beta-glucans with varying molar masses in the context of CD, using an animal model of CD induced by TNBS (2,4,6-trinitrobenzosulfonic acid). Ninety-six Sprague–Dawley rats were divided into control and colitis-induced groups, each further subdivided based on a diet containing standard feed or feed supplemented with low- or high-molar-mass oat beta-glucans for 3 or 7 days, representing acute inflammation and pre-remission stages, respectively. The analysis focused on the gene expression of chemokines and their receptors in the colon wall via RT-PCR and the immunohistochemical analysis of selected proteins in the mucosa. The results indicated that both acute and pre-remission stages of colitis were associated with the increased gene expression of seven chemokines and four chemokine receptors in the colon wall, along with the disrupted protein expression of specific markers in the mucosa. Notably, the consumption of oat beta-glucans, particularly those with a high molar mass, led to a decrease in the expression of most of these genes and modulated the expression of all proteins studied. The high-molar-mass beta-glucan exhibited a stronger effect. In summary, the study suggests that dietary oat beta-glucans, especially those with a high molar mass, have the potential to mitigate colitis by influencing the expression of chemokines, their receptors, and certain proteins associated with CD.

The second in vivo study in the animal model investigates the preventive effects of linseed supplementation (both in as an oil and in extruded forms) on an active mouse model with CD susceptibility. The study focuses on the interplay between dietary factors, physical activity, and the gut microbiota in the context of CD. It is conducted on CEABAC10 transgenic mice fed a high-fat diet (HFD) challenged with adherent–invasive *E. coli* (AIEC) to mimic CD conditions. Two forms of linseed supplementation are examined for their impact on body composition, AIEC-induced intestinal inflammation, and mucosa-associated microbiota. The results indicate that both linseed supplementation forms (oil and extruded) have a beneficial effect on AIEC-induced intestinal inflammation, as evidenced by lower fecal lipocalin-2 concentrations. However, the extruded linseed has a more pronounced impact on mucosa-associated microbiota diversity, increasing the abundance of beneficial bacteria such as *Clostridiales*, *Paraprevotella*, *Prevotella*, and *Ruminococcus*. Additionally, linseed supplementation increases the *Firmicutes*/*Proteobacteria* ratio, indicating a potentially less colitogenic microbiota. Furthermore, the study explores the potential role of linseed in influencing SCFA (short-chain fatty acid) production by the gut microbiota. Butyrate, a major SCFA with anti-inflammatory properties, tends to increase in the group receiving extruded linseed. The study emphasizes the importance of the linseed matrix, including components like lignans, fibers, and other bioactive substances, in modulating the gut microbiota and preventing inflammation. In conclusion, the findings suggest that linseed supplementation, especially in the extruded form, may offer a preventive strategy for managing the chronic inflammation and dysbiosis associated with CD. The study highlights the potential benefits of dietary interventions in influencing the gut microbiota and mitigating inflammatory responses in CD [2].

Another study, a cohort study on CD in the human population, investigates the dietary patterns and nutritional status of individuals with active (CD) in comparison to those in an age-matched group of healthy subjects (HS). The research focuses on the impact of the Western diet, characterized by high animal protein, saturated fats, and processed foods, as a potential risk factor for IBDs. The analysis covers macronutrient intake, dietary patterns, vitamin and mineral consumption, and blood nutrient values. The findings reveal that caloric intakes do not significantly differ between CD and HS groups. CD patients exhibit reduced consumption of vegetables, fiber, dairy, and fish, coupled with increased intake of red meat. Additionally, they show lower levels of vitamins A, E, C, and B6, folic acid, and minerals such as calcium, potassium, phosphorus, iron, magnesium, copper, and iodine. Blood analysis further indicates lower levels of iron, potassium, and total cholesterol in CD patients compared to HS. Amino acid concentrations in the blood are significantly reduced in CD patients, suggesting the potential need for supplementation. In its discussion, this study underscores the impact of CD symptoms on dietary choices, elucidating their potential to lead to the consumption of an unbalanced diet that may affect gut health. The reduced intake of specific nutrients in the blood is associated with impaired absorption, emphasizing the necessity for targeted supplementation. In conclusion, individuals with active CD tend to adopt unbalanced diets influenced by symptomatology, resulting in nutrient deficiencies. This study highlights the critical role of specific nutritional plans and supplements in addressing deficiencies caused by intestinal malabsorption in CD patients [3].

The fourth research article discusses the role of crocetin, a compound found in saffron, in the context of dextran sulfate sodium (DSS)-induced colitis in mice, which is a model for UC. This study introduces the concept that dysbiosis in the intestinal microbiota and an exaggerated immune response to non-pathogenic bacteria may play pivotal roles in UC development. The text underscores the importance of understanding the intricate relationship between the gut microbiota and the host in exploring UC pathogenesis. Crocetin, a major active component of saffron, is discussed in this study for its potential therapeutic properties, including anti-inflammatory, anti-depressant, anti-tumor, anti-oxidant, and hepatoprotective effects. It aims to investigate the effects of crocetin on colon pathology, gut microbiota composition, and microbial metabolites in mice with DSS-induced colitis. The results indicate that, in the colitis mouse model, crocetin intake leads to weight loss, increased intestinal permeability, and an imbalance in the gut microbiota composition. The study delves into specific changes in bacterial genera, such as increased *Akkermansia* and *Mediterraneibacter* and decreased *Muribaculaceae*, *Dubosiella*, *Paramuribaculum*, and others. Furthermore, crocetin treatment is associated with alterations in the gut metabolome, including changes in bile acid metabolites, arachidonic acid, and inflammatory mediators. It suggests that crocetin may exacerbate UC symptoms by affecting the gut microbiota, inducing changes in microbial metabolites, and potentially influencing the arachidonate signaling pathway. In conclusion, the findings highlight the complex interactions between crocetin, gut microbiota, and the host metabolism in the context of colitis [4].

The last research article concerns the anti-cancer effect of ononitol monohydrate (OMH), a glycoside isolated from *Cassia tora*, in terms of its inhibition of HT-115 human colorectal cancer cell proliferation. It has been already observed that OMH is biologically safe, and potentially inhibits inflammatory cytokine development in macrophages, in addition to regulating lipid metabolism in adipocytes. In this paper, the author aimed to suppress pro-tumorigenic inflammatory cytokine COX-2/PGE-2 expression using OMH, further controlling the regulation of cytokine transcription factor and proinflammatory signaling networks, inhibiting CRC progression. The treatment available for IBD or CRC is critical due to the toxicity of COX-2 inhibitors, which pose the potential risk of aggravating colitis and colonic injury; for this reason, the use of a non-toxic natural agent may represent a novelly important beneficial tool. The results obtained showed that OMH exerted cytotoxic activity in HT-115 colorectal carcinoma cells. Its mechanism of action was associated with the cellular uptake of OMH, thus arresting COX-2 expression and further lowering PGE-2 levels by inhibiting arachidonic acid utilization. In addition, OMH stimulates apoptosis via the inhibition of the apoptosis inhibitor NF-kB; taken together, the findings of this article demonstrate that OMH represents a useful agent for the prevention and treatment of CRC [5].

Apart from the five research articles, two reviews were also published in this Special Issue. Jang et al. [6] summarize the efficacy of *Scutellaria baicalensis* Georgi (SBG), one of the medicinal herbs that have been most extensively used in China for over 2000 years, and its flavonoid components (mainly baicalin, baicalein, wogonoside, and wogonin), in IBD and CRC. Many studies have demonstrated the wide range of pharmacological effects of SBG, including anti-inflammatory, anti-viral, anti-bacterial, anti-oxidant, anti-cancer, and immunomodulatory activities, based on the findings of a 2022 human trial that explored the efficacy and safety of a complex herbal extract from the roots of *Pueraria lobata* and SBG in patients with acute ischemic stroke. In detail, the review highlights how the different flavonoids could exert their beneficial properties in IBD and CRC. In IBD, reductions in cyclooxygenases-2 (COX-2) levels, an anti-oxidant enzyme catalyzing the conversion of free arachidonic acid into prostaglandin H2, which plays a significant role in inflammatory and pain responses, represent an efficient strategy of action. In addition, the inhibition of the TLR4/NF-kB pathway, the main orchestrator of the inflammatory response, showed potential therapeutic benefits in the treatment of inflammatory conditions. Important results were also obtained in CRC; several studies showed how SBG could inhibit cancer cell growth both in vitro and in vivo. The mechanisms involved were (i) the promotion of apoptosis, a natural mechanism of programmed cell death that is a highly regulated process that eliminates unnecessary or unwanted cells, to reduce the uncontrolled growth of cancer cells. Strictly correlated with apoptosis is also the (ii) inhibition of the PI3K/AKT/mTOR signaling cascade, which is overexpressed in different forms of cancer, thus representing a significant approach for cancer prevention and treatment. In conclusion, SBG was able to reduce the release of inflammatory factors in several in vitro and in vivo models of IBD, exhibiting anti-cancer activity through the regulation of different pathways.

The second review of the Special Issue provides an overview of the anti-inflammatory and anti-oxidant properties of hydroxytyrosol (HT), the main polyphenol present in olive oil and leaves, against intestinal and gastrointestinal diseases. Arangia et al. (z) underlined the importance of the Mediterranean diet, which is characterized by a regular intake of extra virgin olive oil, the significant beneficial effects of which are attributed to its phenolic constituents, fatty acid composition, and carotenoids such as lutein and beta-carotene. Focusing on HT, the authors properly describe its sources, the main procedures related to its extraction and purification, its bioavailability, and the toxicity related to its consumption. The role of HT in IBDs is strictly related to its well-recognized anti-oxidant and anti-inflammatory properties. In UC, HT has been shown to have protective and immunomodulatory effects against intestinal inflammation in several in vivo and in vitro experimental models in terms of its inhibition of NLRP3 inflammasome activation and its modulation of the NF-kB signaling pathway, the consequent release of pro-inflammatory cytokines, and the expression of molecules downstream of the inflammatory cascade, such as COX-2 and inducible nitric oxide synthase (iNOS). In addition, HT promoted Nrf2 nuclear localization, defending against ROS by inducing anti-oxidant enzyme synthesis. Finally, HT modulated the apoptotic process, downregulating the apoptotic gene Bax and upregulating the anti-apoptotic gene Bcl2. In CD, HT has been defined as a strong anti-oxidant and free radical scavenger, exerting its anti-inflammatory activity through the inhibition of p38/MAPK and NF-kB signaling pathways with a reduction in iNOS expression and nitric oxide release. Furthermore, HT possesses an important role in other gastrointestinal diseases too, such as (i) gastric ulcers, showing beneficial effects against the proliferation of Helicobacter pylori (ii) in CRC, for example, through inhibiting the cell proliferation of colon cancer cells by activating estrogen receptor-β and upregulating anti-oxidant enzymes, and also in (iii) gastroesophageal reflux disease and eosinophilic esophagitis, through protecting gastric mucosa from oxidative damage and its associated complications, mainly via the modulation of the inflammatory response [7].

In conclusion, this Special Issue delves into the promising realm of utilizing dietary bioactive compounds, particularly the phytochemicals abundant in fruits and vegetables, for the prevention and management of IBD. The seven papers, comprising five research articles and two reviews, collectively contribute valuable insights into various facets of IBD, offering potential avenues for the development of therapeutic interventions. The findings underscore the intricate interplay of factors such as dietary components, gut microbiota, and immune responses in the context of IBD. Collectively, these studies not only deepen our understanding of the molecular mechanisms involved in IBD but also illuminate the potential of dietary interventions and bioactive compounds in shaping therapeutic strategies. As the prevalence of IBD continues to rise globally, these findings create the potential for the further research and development of novel, nature-inspired approaches toward IBD prevention and treatment.
